# Successful treatment of deep vein thrombosis caused by iliac vein compression syndrome with a single-dose direct oral anti-coagulant

**DOI:** 10.1186/s12959-017-0128-2

**Published:** 2017-02-01

**Authors:** Naoya Nakashima, Daisuke Sueta, Yusuke Kanemaru, Seiji Takashio, Eiichiro Yamamoto, Shinsuke Hanatani, Hisanori Kanazawa, Yasuhiro Izumiya, Sunao Kojima, Koichi Kaikita, Seiji Hokimoto, Kenichi Tsujita

**Affiliations:** Department of Cardiovascular Medicine, Graduate School of Medical Sciences, Kumamoto University, 1-1-1, Honjo, Chuo-ku, Kumamoto 860-8556 Japan

**Keywords:** Thromboembolism, Iliac vein compression syndrome, Direct oral anti-coagulant

## Abstract

**Background:**

Although vein stenting is popular for treatment for venous thromboembolism due to mechanical compression, some cases are forced to avoid inserting align agents because of immunodeficiency.

**Case presentation:**

An 82-year-old man with left extremity redness and swelling presented to a hospital for a medical evaluation. The patient was immunodeficient because of the adverse effects of his treatment for Castleman’s disease. A contrast-enhanced computed tomography scan revealed a venous thromboembolism in inferior vena cava and the left lower extremity. Magnetic resonance venography showed that the iliac artery was compressing the iliac vein. We were reluctant to place a stent in the iliac vein has because of the patient’s immunodeficient status. Three months of treatment using single-dose edoxaban (30 mg daily) resulted in complete resolution of the thrombus. This is the first report demonstrating that single-dose edoxaban without acute-phase parenteral anticoagulation is effective in the treatment of iliac vein compression.

**Conclusions:**

A single-dose direct oral anti-coagulant without acute-phase parenteral anticoagulation is effective for mechanical compression

## Background

Vein stenting [1] is the generally accepted treatment for venous thromboembolism (VTE) due to mechanical compression, such as Paget-Schroetter syndrome and iliac vein compression syndrome (IVCS). However, in immunodeficient patients, this procedure may not be appropriate. We present a case of IVCS effectively treated only with a single-dose direct oral anticoagulant (DOAC) without acute-phase parenteral anticoagulation.

## Case presentation

**Fig. 1:**
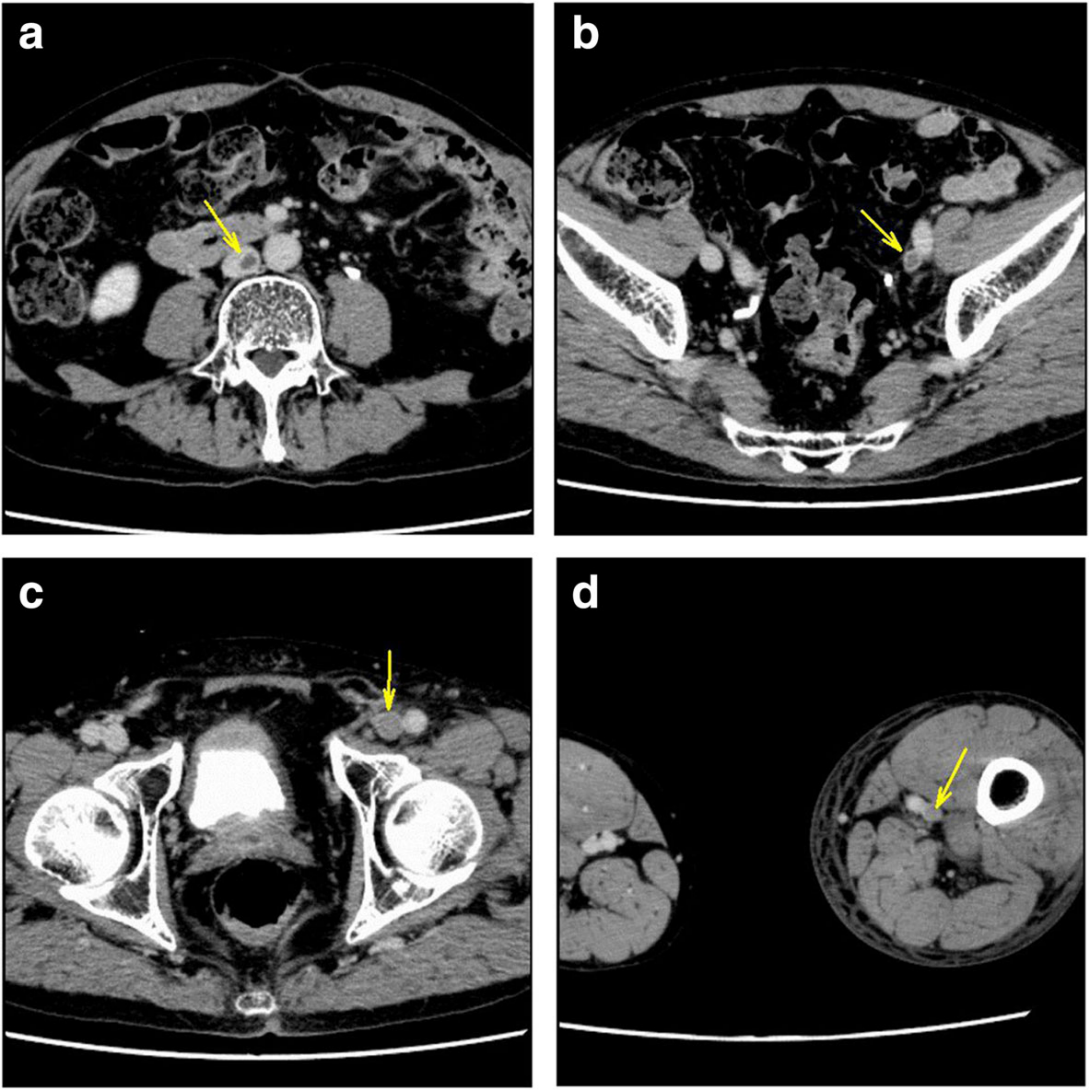
Contrast-enhanced computed tomography on the first visit. A contrast deficit in (**a**) the inferior vena cava at the level of kidney lower lobe (the yellow arrow), (**b**) the left external iliac vein (the yellow arrow), (**c**) the left superficial femoral vein (the yellow arrow), (**d**) the left popliteal vein (the yellow arrow)

**Fig. 2:**
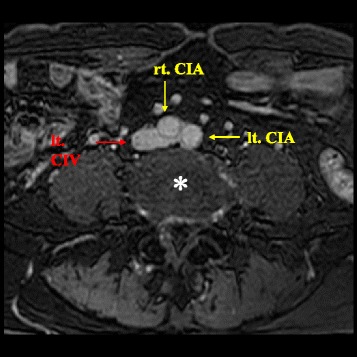
Magnetic resonance venography (T2-TRA) on the first visit. rt. CIA; right common iliac artery, lt. CIA; left common iliac artery, lt. CIV; left common iliac vein, *: vertebral body at fifth lumbar vertebra

**Fig. 3:**
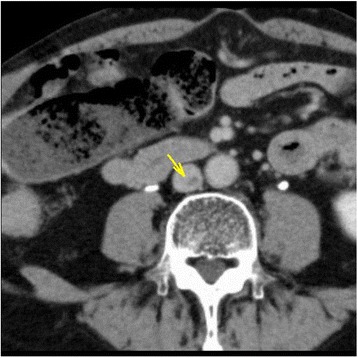
Contrast-enhanced computed tomography at 7 days after the initiation of anticoagulation therapy. The yellow arrow indicates reduced thrombosis

An 82-year-old, nonsmoking man with no history of VTE presented to an emergency department (ED) with a complaint of left lower extremity redness and swelling of 3 days duration. The patient had Castleman’s disease [2, 3], and was being treated with tocilizumab (Actemra™, Chugai Pharmaceutical Co., Ltd.) 370 mg per 3 weeks and predonisolone 4 mg daily [4]. He was also taking acyclovir, voriconazole and sulfamethoxazole-trimethoprim (Baktar Combination Tablets™, Shionogi & Co., Ltd.) for immunodeficiency, a side effects of tocilizumab, in addition to clopidogrel 75 mg daily for a history of cerebral infarction. The serum D-dimer levels in the ED was 5.2 μg/mL, and VTE was suspected. A contrast-enhanced computed tomography (CT)-scan revealed prominent swelling in his left extremity and contrast deficits in the inferior vena cava (IVC) at the level of the lower lobe of kidney (Fig. 1a) and from the left external iliac vein (Fig. 1b) to the left superfacialis femoral vein. There was no evidence of pulmonary embolisms. The patient was diagnosed with VTE and admitted to our department. On admission, his blood pressure was 161/95 mmHg and his heart rate was 63 beats per minute. An arterial blood gas revealed an oxygen saturation (SaO_2_) of 95%. His body mass index was 23.0 kg/m^2^. Anticoaglation therapy, with a DOAC, edoxaban (Lixiana® and Savaysa®, Daiichi Sankyo, Inc.) 30 mg daily was initiated. An IVC filter was not inserted consistent with the latest guideline [5]. Magnetic resonance venography demonstrated that the bifurcation of the left common iliac vein was compressed between the right common iliac artery and the fifth lumbar vertebral body (Fig. 2), confirming a diagnosis of IVCS. A hypercoagulability work-up revealed that protein C, protein S, antithrombin, and antiphospholipid antibodies were within normal limits. After 7 days, a follow-up contrast-enhanced CT scan showed a reduction in the size of the thrombosis (Fig. 3). The patient was permitted to discharge to home.

**Fig. 4:**
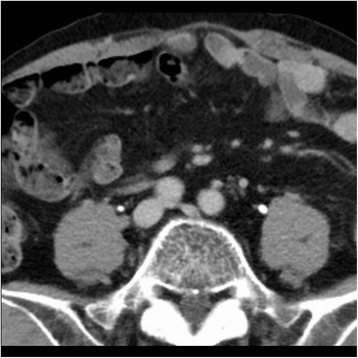
Contrast-enhanced computed tomography at 3 months after the initiation of anticoagulation therapy. The thrombosis was disappeared completely

Anticoagulation therapy with edoxaban was continued for 3 months according to recent guidelines [6], and the VTE was reevaluated. A follow-up contrast-enhanced CT scan revealed that the thrombosis had completely resovled (Fig. 4). Moreover serum D-dimer concentration levels was negative. Edoxaban treatment was then discontinued according to a guideline [7], his clinical symptom, physical examination and D-dimer levels were monitored every month. The patient received treatment with no hemorrhagic complications during clinical course.

The patient gave his consent for the publication of this study.

## Discussion

In 1957, May R and Thurner J reported that 22% of 430 cadavers had stenosis of the left common iliac vein caused by compression of the right iliac artery, with wall thickening of the iliac vein and spur-like formation. The authors speculated that the compressions were due to chronic mechanical compression and out-forces from artery beats [8]. In 1967, using venography, Cockett et al. reported that iliac vein compressions were observed in 65% of VTEs of the left iliac vein, and coined IVCS [9]. In many cases, chronic iliac vein stenosis is asymptomatic because of the development of collateral branches in the pelvic cavity. The diagnosis is usually made when there is greater than 50% stenosis of the iliac vein [10],or over 2 mmHg of pressure gradient in the vein [11]. The first objective in the treatment of mechanical compression is release. Some studies have reported good results with iliac vein stenting for IVCS in both early and chronic phases [12, 13]. Stenting are generally performed overseas. In this case, consistent with the currrent literatures, we considered iliac vein stenting, but were reluctant the insert of align agents because of the patient’s immunodeficient status.

VTEs can result from a variety of causes. Inheritaed thrombophilia, malignant diseases [14, 15], mechanical compression from sleeping in a vehicle after an accident [16], lower limb operations [17, 18], and pregnancy are all important consideration in the differential diagnosis. In this case, thrombus formation was considered to be due to steroid administration, in addition to iliac compression, since steroid hormones have been shown to increase coagulation [19, 20].

In Japan, in addition to warfarin, apixaban [21], rivaroxaban [22] and edoxaban [23] are approved oral anti-coagulation agents for VTEs. The initial treatments for VTEs by apixaban and rivaroxaban requires higher doses, while edoxaban exerts its pharmacological effects at the same dose. Because the patient was at an increased risk of bleeding from the combined use of an anticoagulant with his antiplatelet drug, we selected edoxaban. A daily dose of 30 mg edoxaban was chosen because of his low body weight (less than 60 kg). Regimen of edoxaban in Hokusai-VTE study was an administration of edoxaban following acute-phase parenteral anticoagulation [23].

There is one report of successful treatment of IVCS with rivaroxaban (30 mg → 15 mg daily) [24]. This case report adds to the literature by demonstrating that single-dose edoxaban (30 mg daily) without acute-phase parenteral anticoagulation is also effective though long-term follow-up is still required.

Although whether edoxaban treatment continuation or discontinuation was questionable, steroid therapy was already stopped, we judged that his VTE was reversible. However we monitored his clinical symptom, physical examination and D-dimer levels every month. A long term observation should be mandatory.

## Conclusion

A use of single-dose DOAC is well known to be a treatment for VTE [23]. To the best of our knowledge, this is the first report of successful treatment for IVCS with a single-dose DOAC without acute-phase parenteral anticoagulation.

## Abbreviations

CT: Computed tomography
IVC: Inferior vena cava
IVCS: Iliac vein compression syndrome
VTE: Venous thromboembolism
